# Target-organ damage and cardiovascular complications in hypertensive Nigerian Yoruba adults: a cross-sectional study

**DOI:** 10.5830/CVJA-2012-021

**Published:** 2012-08

**Authors:** OO Oladapo, L Salako, L Sadiq, K Shoyinka, K Adedapo, AO Falase

**Affiliations:** Department of Medicine, Cardiovascular Unit, University College Hospital, and Department of Anatomy, College of Medicine, University of Ibadan, Nigeria; University of Ibadan, Nigeria; Country office, World Health Organisation, Lagos Nigeria; Primary Health Egbeda Local Government, Oyo State, Nigeria; Department of Chemical Pathology, College of Medicine, University of Ibadan, Nigeria; Department of Medicine, Cardiovascular Unit, University College Hospital, College of Medicine, University of Ibadan, Nigeria

**Keywords:** target-organ damage, left ventricular hypertrophy, microalbuminuria, retinopathy, established cardiovascular disease, hypertension, Nigeria

## Abstract

**Background:**

Hypertension is a major challenge to public health as it is frequently associated with sudden death due to the silent nature of the condition. By the time of diagnosis, some patients would have developed target-organ damage (TOD) and associated clinical conditions (ACC) due to low levels of detection, treatment and control. TOD and ACC are easy to evaluate in a primary healthcare (PHC) setting and offer valuable information for stratifying cardiovascular risks in the patient. The aim of this study was to evaluate the prevalence and correlates of TOD and established cardiovascular disease (CVD) in hypertensive Nigerian adults.

**Methods:**

A cross-sectional study was conducted on 2 000 healthy Yoruba adults between 18 and 64 years who lived in a rural community in south-western Nigeria. Participants diagnosed to have hypertension were examined for TOD and ACC by the presence of electrocardiographically determined left ventricular hypertrophy (LVH), microalbuminuria or proteinuria, retinopathy, or history of myocardial infarction and stroke.

**Results:**

A total of 415 hypertensive participants were examined and of these, 179 (43.1%) had evidence of TOD and 45 (10.8%) had established CVD. TOD was associated with significantly higher systolic (SBP) and diastolic blood pressure (DBP). The prevalence of LVH was 27.9%, atrial fibrillation 16.4%, microalbuminuria 12.3%, proteinuria 15.2%, hypertensive retinopathy 2.2%, stroke 6.3%, congestive heart failure (CHF) 4.6%, ischaemic heart disease 1.7%, and peripheral vascular disease 3.6%. Compared with those with normal blood pressure (BP), the multivariate adjusted odds ratios (95% confidence interval) of developing TOD was 3.61 (0.59–8.73) for those with newly diagnosed hypertension; 4.76 (1.30–13.06) for those with BP ≥ 180/110 mmHg; and 1.85 (0.74–8.59) for those with diabetes mellitus.

**Conclusions:**

This study provides new data on TOD and its correlates in a nationally representative sample of hypertensive adults in Nigeria. In this low-resource setting, attempts should be made to detect hypertensive patients early within the community and manage them appropriately before irreversible organ damage and complications set in. The methods used in this study are simple and adaptable at the primary healthcare level for planning prevention and intervention programmes.

## Abstract

Hypertension is often asymptomatic and recording blood pressure is opportunistic. Many of these patients are unaware of their condition and therefore remain untreated. Untreated or poorly controlled hypertension and left ventricular hypertrophy (LVH) are risk factors for cardiovascular diseases (CVD),^1^ a major cause of morbidity and mortality, and sudden death.^2^ The lack of awareness of the disease results in worse outcomes.

A number of hypertensives may present for the first time with target-organ damage (TOD) involving various organs. Therefore at initial diagnosis, they already have hypertensive heart disease (HHD); some with LVH, while some have frank congestive heart failure (CHF). CHF is a lethal disease with a poor prognosis and reduced life expectancy.^3^ It imposes a large health, economic and social burden on the patient, his/her family and the community at large, and it usually affects males and females in their productive middle years.

Major TOD of hypertension such as LVH,^4^ diastolic dysfunction,^5^ CHF,^6^ ischaemic heart disease (IHD),^7^ stroke^8^ and renal failure^9^ have been documented by various workers in Nigeria and these were mostly hospital-based studies. Autopsy studies confirmed that the commonest cause of sudden, unexpected death in Nigerians is hypertension.^10^,^11^

In Nigeria, little is known about the experiences of caregivers and care-seeking practices with regard to hypertension and factors influencing late presentation with TOD. Some of the undiagnosed hypertensives are detected incidentally during the course of management for other clinical conditions or during catastrophic events. The challenge to the physician therefore is early detection and treatment of hypertension before progression to complications of TOD, which portends a poor prognosis.

There are few studies on target-organ involvement in hypertensive Nigerian patients at the secondary and tertiary level of care,^12^-^14^ but few or none in the general population and in the primary-care setting. The aim of our study was to evaluate the prevalence of TOD and established CVD in a hypertensive adult Nigerian population, using methods that could easily be adopted at the primary health centre (PHCs) level. This would be helpful to the health planners for formulation and implementation of preventative strategies.

## Methods

This work was part of a community-based, descriptive, non-interventional, cross-sectional survey of cardiometabolic risk factors, conducted from December 2002 to November 2005 in the Egbeda local government area (ELGA), a rural community in south-western Nigeria, with a population of 128 000. The local governments are serviced by PHCs which are meant to serve as the initial point of care for the majority of patients in Nigeria. The study protocol was evaluated and approved by the Ethics of Human Research Committee of the State Ministry of Health. Individual consent was obtained verbally and where possible by written consent.

Four hundred and fifteen confirmed hypertensive adults (184 males and 231 females) above 18 years of age who participated in the cross-sectional, community-based survey formed the population for this study. Socio-demographic and anthropometric data were collected for each subject. Self-reported and patient clinical records of established CVD and complications such as heart failure, stroke, myocardial infarction, angina, peripheral vascular disease and kidney disease were recorded.

Clinic blood pressure (BP) was measured by trained health workers in each PHC, according to guidelines of the International Society of Hypertension (ISH)/World Health Organisation (WHO) 1999 and the JNC-7.^15^,^16^ Measurements were taken using a standard mercury Accoson sphygmomanometer (Accoson works, Vale Road, London N4 1PS) with appropriate cuff size. Three BP measurements were taken using the subject’s right arm with the subject in the sitting position after five minutes of rest, with one minute between measurements. The mean of three measurements was used as the final value.

Participants with an elevated BP measurement were invited to attend a second clinic visit after one to two weeks to have their BP measured again. The average BP of the second visit was used as a criterion for the diagnosis and control of hypertension. In addition, all treated hypertensive patients were asked to return for a second visit after one to two weeks to have their BP measured.

Hypertension was defined as systolic blood pressure (SBP) ≤ 140 mmHg, diastolic blood pressure (DBP) ≤ 90 mm Hg, or current treatment with antihypertensive drugs in subjects with a history of hypertension.^16^,^17^ Awareness of hypertension meant a previous diagnosis of hypertension or high blood pressure. Controlled hypertension was defined as treated hypertension with SBP < 140 mmHg and DBP < 90 mmHg at the second clinic visit.

Each subject underwent further physical examination to determine clinical features of LVH, CHF, stroke, renal failure, and fundoscopy for hypertensive retinopathy. Hypertensive cardiac damage was defined by the presence of electrocardiographic (ECG) LVH based on the voltage criteria of Araoye^18^ in black hypertensives. LVH was determined as follows: the sum of SV_2_ + RV_6_ in males of ≥ 40 mm and females of ≥ 35 mm and RI ≥ 12 mm. These criteria have been shown to correlate better with echocardiograhic LVH in Nigerians.^19^ Hypertensive eye damage was diagnosed based on the Keith-Wagener classification of hypertensive retinopathy and the patients were divided into four grades.^20^

Renal damage was diagnosed based on the presence of microalbuminuria as determined by spot urine albumin-to-creatinine ratio [ACR (mg/g)]. In males, ACR < 2.5 mg/g is normal, 2.5–25 mg/g defines microalbuminuria and > 25 mg/g indicates gross proteinuria. In females, ACR < 3.0 mg/g is normal, 3.0–30 mg/g defines microalbuminuria, and > 30 mg/g indicates gross proteinuria.^21^-^24^

Venous blood samples were obtained via the ante-cubital vein for biochemical assessment, including fasting serum total cholesterol, high-density lipoprotein cholesterol (HDL-C) and fasting blood glucose levels.

## Statistical analysis

The data obtained were analysed using SPSS version 13.0 software (SPSS Inc, Chicago, Illinois, USA). Descriptive analysis of the variables was performed to process the data as tables. Continuous variables were described by calculating the means and standard deviation (SD). Categorical variables were described using frequency tables.

## Results

A total of 415 subjects consisting of 184 men (44.3%) and 231 women (55.7%) participated in the study. The baseline characteristics of the subjects are shown in Table 1. The females were significantly older [50.4 (± 13.2) years] than the males [46.9 (± 16.7) years] (*p* < 0.001). SBP and DBP did not vary significantly according to gender. The detection and treatment rate of hypertension was low in the studied population. Only 14.2% of the subjects had self-reported hypertension and of these, only 18.6% had been on medications in the past three months; of whom only 27.3% had controlled blood pressure. This means that only 5.1% of subjects who had self-reported hypertension had controlled BP at the time of the study.

**Table 1.** Characteristics Of The Subjects Included In The Study

	*Males (n = 184) Mean (SD)*	*Females (n = 231) Mean (SD)*	*Total (n = 415) Mean (SD)*
Age (years)	43.4 (± 20.2)	50.4 (± 13.2)	46.9 (± 16.7)
BMI (kg/m^2^)	25.1 (± 4.8)	28.7 (± 6.5)	26.9 (± 5.7)
Waist circumference (cm)	86.5 (± 14.7)	92.6 (± 13.3)	89.5 (± 14.0)
SBP (mmHg)	159.4 (± 25.5)	156.9 (± 24.2)	158.2 (± 24.9)
DBP (mmHg)	98.7 (± 13.6)	94.9 (± 17.5)	96.8 (± 15.4)
Level of education			
None	(28) 15.2%	(59) 25.5%	(87) 21.0%
Primary	(101) 54.9%	(136) 58.9%	(237) 57.1%
Secondary	(43) 23.4%	(31) 13.4%	(74) 17.8%
Tertiary	(12) 6.5%	(5) 2.2%	(17) 4.1%
Current cigarette smoker	(21) 11.4%	(0) 0.0%	(21) 5.1%
Current tobacco use*	(0) 0.0%	(3) 1.3%	(3) 0.7%
Diabetes	(13) 7.1%	(27) 11.7%	(40) 9.6%
Hypercholesterolaemia			
Hypertension (≥ 140/90 mmHg)	(19) 10.3%	(22) 9.5%	(41) 9.9%
Newly diagnosed	(163) 88.6%	(193) 83.5%	(356) 85.8%
Self-reported	(21) 11.4%	(38) 16.5%	(59) 14.2%
Receiving treatment in past			
3 months	(4) 19.0%	(7) 18.4%	(11) 18.6%
BP control	(1) 25.0%	(2) 28.6%	(3) 27.3%

*Tobacco use (chewing/snuffing).

The antihypertensive drugs used either alone or in combination included centrally acting alpha-adrenergic agonists such as α-methyl DOPA and Brinerdin (74.3%), thiazide diuretics (86.1%), calcium channel blockers (15.6%) and ACE inhibitors (1.7%). Some of the subjects who self-reported hypertension were on benzodiazepines prescribed as antihypertensives by their general practitioner.

The prevalence of diabetes among the participants was 9.6% (7.1% in men and 11.7% in women). The prevalence of smoking in the study population was 5.1% and all the cigarette smokers were men.

Table 2 shows the prevalence of hypertensive TOD among the subjects studied. Overall, 179 (43.1%) of the subjects, 83 (20.0%) men and 96 (23.1%) women, had evidence of TOD. The age difference between those subjects with (47.4 ± 11.5 years) and without (45.9 ± 13.2 years) TOD was not statistically significant (*p* = 0.12). The blood pressure of subjects in relation to TOD is shown in Table 3. Subjects with TOD had significantly higher (*p* < 0.001) mean SBP (167.1 ± 15.6 mmHg) than those without TOD (156.3 ± 18.7 mmHg). Mean DBP was also significantly higher (*p* < 0.001) in those with TOD (102.3 ± 9.5 mmHg) than those without (95.8 ± 13.5 mmHg).

**Table 2.** Prevalence Of Hypertensive Target Organ Damage Among Participants

*Target-organ damage*	*Number (n = 415)*	*Prevalence (%)*
Heart LVH	116	27.9
LAE	91	21.9
Atrial fibrillation	68	16.4
Old infarcts/ischaemic changes	51	12.3
Retinopathy		
grade 0	83	20.0
grade 1	167	40.2
grade 2	156	37.6
grade 3	9	2.2
grade 4	0	0.0
Renal damage*		
Microalbuminuria	51	12.3
Gross proteinuria	63	15.2

LVH = left ventricular hypertrophy; LAE = left atrial enlargement; *Men, microalbuminuria = albumin-creatinine ratio (ACR) 2.5–25 mg/g and gross proteinuria is ACR > 25 mg/g; women, microalbuminuria = ACR 3.0–30 mg/g and gross proteinuria is ACR > 30 mg/g.

**Table 3.** Blood Pressure Ranges Of Subjects In Relation To Target-Organ Damage

	*Blood pressure (mmHg)*			
*Target organ*	*SBP (± SD)*	*DBP (± SD)*	p-*value*
ECG abnormalities’			
Present	161.7 ± 18.9	99.4 ± 9.5	
Absent	153.3 ± 16.2	91.5 ± 10.1	< 0.001
Retinopathy			
Present	155.6 ± 15.4	93.7 ± 12.1	
Absent	152.8 ± 18.2	95.2 ± 13.0	0.52
Renal damage			
Present	163.5 ± 17.6	101.6 ± 9.5	
Absent	150.0 ± 16.2	90.9 ± 9.5	0.001

The prevalence of LVH was 27.9% and atrial fibrillation was 16.4%. Subjects with LVH had significantly higher (*p* < 0.001) SBP (161.7 ± 18.9 mmHg) compared to those without LVH (153.3 ± 16.2 mmHg) and their DBP was also significantly higher (*p* < 0.001) at 99.4 ± 9.5 mmHg, compared with 91.5 ± 10.1 mmHg.

Grade 3 hypertensive retinopathy was present in 2.2% of the study population, and the commonest retinopathy was grade 1 (40.2%), followed by grade 2 (37.6%). Microalbuminuria was present in 12.3% and gross proteinuria in 15.2%. The established CVD found in the subjects is shown in Table 4. History of stroke was found in 6.3% and CHF in 4.6% of the participants. Ischaemic changes and evidence of old myocardial infarcts were seen on ECG in 12.3% of the participants. However, only 0.5% gave a history of myocardial infarction and 1.2% gave a history of angina. Peripheral vascular disease was found in 3.6% of the subjects, of whom 73.3% (11/15) were diabetic. Overall, 45 (10.8%) subjects had established CVD.

**Table 4.** Prevalence Of Established Cardiovascular Disease Among Participants

*CV complications*	*Number (n = 415)*	*Prevalence (%)*
Stroke	26	6.3
Myocardial infarction	2	0.5
Angina	5	1.2
Congestive heart failure	19	4.6
Peripheral vascular disease	15	3.6

The results of multiple regression analyses after adjusting for potentially confounding variables are presented in Table 5, showing the association between TOD and selected variables. The odds of developing TOD were increased by new diagnosis of hypertension, systolic blood pressure ≥ 180 mmHg/diastolic blood pressure ≥ 110 mmHg, and diabetes mellitus.

**Table 5.** Association Between Hypertensive Target-Organ Damage And Selected Variables

Detection of hypertension	OR* (95% CI) for target-organ damage
Self-reported	1.00
Newly diagnosed	3.61 (0.59–8.73)
BMI	
≤ 24.9 kg/m^2^	1.00
25.0–29.9 kg/m^2^	0.95 (0.28–3.26)
≥ 30.0 kg/m^2^	1.44 (0.45–5.74)
Hypertension classification	
BP < 140/90 mmHg	1.00
SBP 140–159 mmHg/DBP 90–99 mmHg	1.34 (0.23–2.95)
SBP 160–179 mmHg/DBP 100–109 mmHg	1.29 (0.20–4.95)
SBP > 180 mmHg/DBP > 110 mmHg	4.76 (1.30–13.06)
Diabetes mellitus	
No diabetes	1.00
Diabetes	1.85 (0.74–8.59)

*OR (odds ratio) adjusted for age, gender, smoking, level of education, blood pressure control. BMI = body mass index.

## Discussion

The main findings of this study were the following: (1) The presence of TOD was frequent in this rural adult population; (2) The peak age for incidence of TOD was around 47 to 50 years, that is middle age; (3) TOD was related to the presence of newly diagnosed hypertension, stage 3 SBP and DBP, and diabetes mellitus; (4) The presence of established CVD was relatively frequent in this population; (5) There was a low detection rate, treatment and control of hypertension; (6) Atrial fibrillation was found in 16.4% of the population and this may have increased the risk of stroke as no patients were on treatment; (7) In spite of the low levels of angina and myocardial infarction, ischaemic changes and evidence of old infarcts were found in the ECGs of 12.3% of the population studied.

The present study contributes to our knowledge on TOD in Nigerian hypertensives in several ways. First, our study demonstrates an incidence rate of TOD of 43.1% and established CVD of 10.8% among a nationally representative sample of the adult population of hypertensive Nigerians. Most studies of this nature have been conducted in hypertensive Nigerian patients attending healthcare facilities.

The relatively high prevalence of TOD among the community of rural-dwelling adults is worrisome as it approaches previously reported hospital-based values of 53.3%^13^ and 60.1%.^12^ Blacks have been shown to have more severe forms of hypertension with greater risk of TOD.^25^,^26^ This may be due to the fact that 85.8% of the subjects were newly diagnosed hypertensives.

Some of the subjects had had their blood pressure measured for the first time during the study. Of those who had had previous blood pressure measurements, the definition of hypertension by their attending medical team was 160/95 mmHg. These physicians had failed to commence appropriate management even at this level. Furthermore, only 18.6% of the self-reported hypertensives were on any form of treatment, which was mostly suboptimal. Of these, only 27.3% had their blood pressure controlled. There is an urgent need for training and retraining of both the care givers and care seekers at the community and PHC levels in order to reduce the burden of CVD in the population.

The prevalence of ECG-LVH was nearly 28%, based on Araoye’s criteria,^18^ indicating that these individuals had more severe LVH with a possibly worse prognosis. This may have been due to a long duration of undiagnosed hypertension in the subjects. LVH has been associated with increased incidence of CHF, coronary artery disease, stroke, arrhythmias and sudden death.^27^ In those receiving treatment for hypertension, the antihypertensives being used were inadequate to regress their LVH. LVH places the subjects at risk of developing adverse cardiovascular events. The percentage of subjects with LVH was lower than the 31.0%^12^ and 43.3%^13^ obtained in previous studies, although these were hospital based in urban settings. Another study in hypertensive urban civil servants in Ghana found a prevalence of 33.3%.^28^

Left atrial enlargement (LAE) was present on the ECGs of 21.9% of the subjects and atrial fibrillation in 16.4%. These are known risk factors for thrombo-embolic strokes and in the presence of hypertension, they may worsen the prognosis. Hypertension, a major risk factor for stroke in sub-Saharan Africa, including Nigeria, has been reported to occur in 33 to 62% of those with cerebral infarction and 60 to 92% of those with cerebral haemorrhage.^29^

The relatively high prevalence of stroke in our study population was 6.3% and this was comparable to a hospital-based report of 8.9%^12^ but much higher than the 0.8% reported in a similar setting.^13^ In sub-Saharan Africa, stroke is a major CVD of public health concern, with high morbidity and mortality, affecting people in the prime of their lives.^30^,^31^ It also imposes a high economic burden on the healthcare systems, which this low-income country cannot afford.

Kidney damage was found in 27.5% of the subjects and of these, 12.3% had microalbuminuria, while 15.2% had gross proteinuria. There is disparity between our findings and a previous study that reported microalbuminuria in 37% and overt proteinuria in 2% of newly diagnosed Nigerian hypertensives.^32^ Microalbuminuria is a sensitive marker for renal damage in hypertension.^16^

In our study, subjects who had ECG abnormalities and evidence of kidney damage had statistically significantly (*p* < 0.001) higher SBP and DBP compared with subjects who had neither TOD. In a study on TOD in hypertensive Ghanaian civil servants,^28^ trace proteins were found in 8.8%, proteinuria in 13.4% and chronic kidney disease in 4% of the participants. The high rate of proteinuria in our study population was a cause of concern as efforts in the primary prevention of chronic kidney disease should be intensified in this low-resource setting. Failure to do so will increase the economic burden on the fragile healthcare system.

Based on the fact that grades 3 and 4 hypertensive retinopathy are used as evidence for TOD,^31^ the prevalence of hypertensive retinopathy in our study was 2.2% and was comparable to the prevalence reported in studies in sub-Saharan Africa.^28^,^32^,^33^ These studies reported a rarity of hypertensive retinopathy in this region, unlike the high prevalence in economically more advanced nations.^34^ About 78% of our subjects had either grade 1 or grade 2 retinopathy.

Of the participants in this study, 10.8% had established CVD. Of these, 6.3% had stroke, 4.6% had CHF, 3.6% had peripheral vascular disease and 1.7% had ischaemic heart disease. Hypertension plays a significant role in the causation of CHF in Nigeria.^12^ Heart failure is another complication which would impose a social and economic burden on the populace. This underscores the importance of establishing intervention programmes for primary prevention, early detection and appropriate management of hypertension in this rural population. Although the prevalence of ischaemic heart disease was low in this study, its presence in the population may be significant, with epidemiological transition occurring at a rate faster than previously thought.

The odds of developing TOD were highest in participants with blood pressure ≥ 180/110 mmHg, followed by newly diagnosed hypertensives and those with diabetes. Previous studies have shown a more positive correlation of hypertensive TOD with SBP than with DBP.^12^,^35^ Since hypertension is a silent disease, there is a need to create awareness to encourage opportunistic screening. Patients should be encouraged to adopt healthy lifestyles for the primary prevention of CVD as they may be unwilling to commence taking drugs, which may have side effects. This is particularly so when they do not have any symptoms.

Of the few patients who were on antihypertensives, the drugs most frequently used were centrally acting ones such as α-methyl DOPA, or inappropriate ones such as benzodiazepines (valium, lexotan). Invariably, patients on such treatments will have uncontrolled BP and are likely to have more TOD and established CVD, with worse outcomes. Newly diagnosed hypertensives should have their cardiovascular risks fully assessed and appropriately managed. Since stage 3 hypertension was associated with significantly increased odds of developing TOD, efforts should be made to aggressively lower and control BP in such individuals.

Our study had some limitations. The investigations did not include echocardiography for assessment of LVH, which is more sensitive than ECG. However, we used locally validated Araoye’s criteria to determine LVH with ECG, as this was likely to give us a more accurate diagnosis.^18^,^19^ The duration of hypertension could not be ascertained in most of our subjects as many had not had their BP checked until the time of the study. Therefore, we could not control for the duration of hypertension, which is a confounder in this type of study.

## Conclusion

This study showed a high level of TOD and established CVD in this rural population of hypertensives. Our findings strongly suggest that the epidemiological transition from communicable to non-communicable diseases is occurring at a faster rate than previously envisaged. In this low-resource setting, there is an urgent need for primordial and primary prevention of hypertension and its complications. Once detected, BP should be properly controlled with appropriate medications. To this effect, both the populace and health practitioners should receive health education and health promotion to reduce the burden of disease.
